# Efficacy of small-incision lenticule extraction surgery in high astigmatism: A meta-analysis

**DOI:** 10.3389/fmed.2022.1100241

**Published:** 2023-01-19

**Authors:** Ge Cui, Yu Di, Shan Yang, Di Chen, Ying Li

**Affiliations:** ^1^Department of Ophthalmology, Peking Union Medical College Hospital, Chinese Academy of Medical Sciences & Peking Union Medical College, Beijing, China; ^2^Key Laboratory of Ocular Fundus Diseases, Chinese Academy of Medical Sciences & Peking Union Medical College, Beijing, China

**Keywords:** small-incision lenticule extraction, high astigmatism, vector analysis, corneal aberrations, meta-analysis

## Abstract

**Purpose:**

This study aimed to evaluate the efficacy of small-incision lenticule extraction (SMILE) in improving vision and visual quality and correcting astigmatism for the treatment of high astigmatism.

**Methods:**

Eligible studies and studies comparing the efficacy of SMILE with femtosecond laser-assisted laser *in situ* keratomileusis (FS-LASIK) or transepithelial photorefractive keratectomy (T-PRK) for high astigmatism (≥2.00 D) were identified in PubMed, Embase, and the Cochrane Central Register of Controlled Trials, searched from their inception to 29 May 2022. The references of all searched studies were checked as supplements. The risk of bias was evaluated for each eligible study. The literature was screened according to the inclusion and exclusion criteria, and relative data were extracted. Data were extracted and analyzed by ReviewManager 5.4. The primary outcome was post-operative uncorrected distance visual acuity (UDVA). The secondary outcomes included corneal aberrations and vector parameters. The weight mean difference (WMD) and their 95% confidence intervals (95% CI) were used to assess the strength of the association.

**Results:**

A total of six studies including 380 astigmatism eyes were involved, with 211 high-astigmatism eyes and 31 low-astigmatism eyes undergoing SMILE surgery, 94 high-astigmatism eyes undergoing FS-LASIK surgery, and 44 high-astigmatism eyes undergoing T-PRK surgery. Compared with non-SMILE, SMILE induced more astigmatism (weighted mean difference [WMD] = −0.07, 95% CI [−0.12 to −0.02], *P* = 0.005) and fewer sphere aberrations (WMD = −0.12, 95% CI [−0.17 to −0.08], *P* < 0.00001). The post-operative UDVA, sphere, spherical equivalent (SE), and higher order aberrations in different surgeries were likewise equivalent. The difference vector and index of success were significantly higher, and the surgically induced astigmatism vector, correction index, and magnitude of error were significantly lower in SMILE.

**Conclusion:**

This meta-analysis suggests that SMILE, FS-LASIK, and T-PRK show excellent efficacy, predictability, and safety for myopia. SMILE exhibited less astigmatism refraction predictability and less surgically induced spherical aberrations. There may be more under-correction in SMILE. More randomized, prospective, and large sample-sized studies are needed to confirm these conclusions in the long term.

## 1. Introduction

Currently, small-incision lenticule extraction (SMILE), femtosecond laser-assisted laser *in situ* keratomileusis (FS-LASIK), and transepithelial photorefractive keratectomy (T-PRK) are the main corneal refractive surgeries. SMILE surgery has been the most common refractive surgery since its inception in 2008 (1). In comparison to other surgeries (2, 3), SMILE surgery is flapless, stable, less invasive, and less painful because it maintains more corneal nerve fibers (4) and causes less biochemical changes (5) in the cornea. Studies (6, 7) have demonstrated that SMILE surgery has good long-term safety and great visual results.

In refractive surgery, the correction of astigmatism is just as crucial as the correction of spherical equivalent (SE), and proper astigmatism correction is essential to achieve optimal visual quality. Since astigmatism is a vector parameter whose quantity and axis must be equally taken into account while designing the refractive surgery, precisely locating and correcting the vector is essential to effectively treat astigmatism. Vector analysis was advised for assessing the effectiveness of astigmatism correction, with the Alpins technique (8) being the most popular. In a few studies (9–12) of SMILE surgery using this technique, slight under-correction and axis misalignment were noticed. This condition may be caused by the surgeon using a subjective centration technique without an eye tracking system and by a lack of cyclotorsion control because a negative pressure suction ring is used to draw the eye, which causes position-related cyclotorsion when the patient’s position changes (13, 14). Few studies (12, 13) have evaluated the effectiveness of SMILE surgery in treating high astigmatism, despite the fact that several studies (12, 13) have examined the results of the procedure in treating low to moderate astigmatism. FS-LASIK surgery appears to have better results for treating low to moderate astigmatism than SMILE (12, 15) surgery, and SMILE surgery appears to be more effective than stromal ablation surgery like photorefractive keratectomy (PRK) (16, 17). We are aware of only a few studies that compare the effectiveness of SMILE with other operations for treating high astigmatism.

Therefore, our goal was to compile all relevant data and to assess the effectiveness of three distinct procedures (viz, SMILE, FS-LASIK, and T-PRK), using vector analysis, for treating high astigmatism of ≥2.00 D. The visual and refractive outcomes as well as corneal wavefront aberrations were also analyzed. We consider that this study could be regarded as supporting well for the decision-making of ophthalmologists for the selection of the refractive surgery method for high-astigmatism eyes. In addition, the corneal wavefront aberrations and the visual and refractive results were examined. This research was considered to be useful in helping ophthalmologists choose the best refractive surgery technique for high-astigmatism eyes.

## 2. Methods

This meta-analysis was performed in accordance with the Preferred Reporting Items for Systematic Reviews and Meta-Analyses (PRISMA) statement (18).^1^

### 2.1. Search strategy

Reports of randomized clinical trials (RCTs) comparing SMILE and FS-LASIK or T-PRK for high astigmatism (≥2.00 D) were identified through a systematic search in PubMed, Embase, Cochrane Central Register of Controlled Trials, Wanfang data, and CNKI from the databases’ inception to 30 June 2022. The keywords were “laser corneal surgery,” “astigmatism,” “vision, ocular,” “night vision,” “corneal wavefront aberration,” and “vector analysis,” with the following Medical Subject Headings (MeSH) as their counterparts (Supplementary material, available in the online version of this article). The references of relevant studies identified using the bibliographic database were also reviewed to identify other potentially related articles. Following the PICOS principle, the key search terms included (P, participants) patients with moderate to high myopia; (I, interventions) patients treated by SMILE or other refractive surgeries; (C/O, comparison/outcome) the comparison of the clinical outcomes; and (S, study design) designed as a clinical cohort study. Only studies on human beings were considered, and there was no language restriction. Full copies of all relevant studies were obtained and assessed to determine whether they met standard quality criteria for inclusion in the study.

### 2.2. Study selection criteria

Inclusion criteria were listed as follows: (1) information from findings on the effect of SMILE surgery on high astigmatism; (2) at least one of the following outcomes provided both pre-operatively and post-operatively: uncorrected distance visual acuity (UDVA), corneal aberrations, and vector parameters; and (3) only studies with vector analysis of astigmatism were selected and included.

Exclusion criteria were listed as follows: (1) patients without high astigmatism; (2) insufficient data to estimate a weighted mean difference (WMD); (3) review articles or technical notes; and (4) redundant publications.

Electronic database citations were collected into an EndNote library. After duplicate citations were removed, the library was imported into Covidence. Citation titles and abstracts were evaluated independently by two reviewers (GC and YD), who then judged whether they should be included, excluded, or potentially included. Studies that were determined to be unquestionably or maybe eligible moved on to full-text screening. Based on pre-established qualifying criteria, two reviewers (GC and SY) independently assessed the full-text submissions. The main justification for studies being eliminated at the full-text screening stage was recorded. Any differences between review authors were settled by discussion or, if necessary, by contacting the third author (DC or YL).

### 2.3. Data extraction and quality assessment

In our study, two reviewers (GC and YD) performed data extraction independently. Results were compared and any disparity between the two reviewers’ results was resolved by discussion. If agreement still could not be reached by the two reviewers, a third reviewer (DC) would assess the data and make the final conclusion. For each study, the following data were extracted: first author, year of publication, sample size (number of eyes), participant’s age, follow-up duration, UDVA, aberration outcomes including spherical aberration (SA), coma, higher-order aberrations (HOAs), and vector parameters, including target induced astigmatism (TIA) vector, surgically induced astigmatism (SIA) vector, difference vector (DV), correction index (CI, SIA/TIA), the magnitude of error (ME, the arithmetic difference between the magnitudes of the SIA and TIA), and index of success (IOS, DV/TIA). UDVA was estimated as ETDRS letter scores, which were transferred into log MAR units according to the formula log MAR = 85 + 50 × log (Snellen fraction) (19). The data from the last follow-up visit in each study were included for analysis.

In addition, two reviewers (GC and SY) independently assessed the included research with the Cochrane Collaboration’s “Risk of Bias” tool from the *Cochrane Handbook for Systematic Reviews of Intervention* (20). The items of evaluation comprise random sequence generation and allocation concealment (selection bias), blinding of participants and personnel (detection bias), incomplete outcome data (attrition bias), selective reporting (reporting bias), and other biases by three different grades (low, high, or unclear) for each article. The certainty of the body of evidence was assessed as high, moderate, low, or very low, for the primary and secondary outcomes using the Grading of Recommendations, Assessment, Development and Evaluation (GRADE) approach (21).

### 2.4. Statistical analysis

Cochrane ReviewManager (RevMan version 5.4) was used to conduct meta-analyses. For the continuous outcomes, weighted mean differences (WMDs) and their 95% CIs were calculated. Heterogeneity was evaluated using the *Q*-test and *I*^2^ tests. An *I*^2^ value of >50% or a *p*-value for Cochrane’s Q statistic of <0.05 was used to define significant statistical heterogeneity (22, 23). The fixed-effects model was employed, unless statistical heterogeneity was significant, in which case a random-effects model was used (24, 25). A *P*-value of less than 0.05 was considered statistically significant. As for indicators for which it was not possible to perform a meta-analysis, a descriptive analysis of outcomes in each study was listed. As there were fewer than 10 trials for each of the intervention categories, funnel plots were not visually inspected to assess small study effects.

## 3. Results

### 3.1. Overview of included studies

**FIGURE 1:**
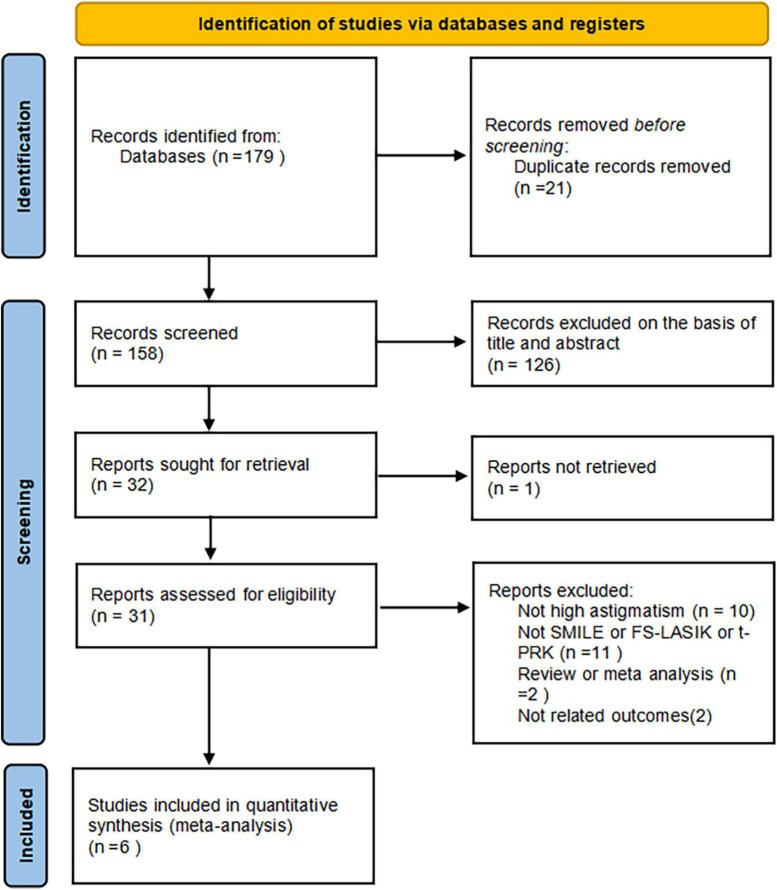
Flow diagram of the identification and inclusion of eligible studies.

A total of 179 potentially relevant articles were identified. Among these studies, 14 duplicates were removed by Endnote software. After screening titles, abstracts, and full texts, six studies were finally included, all of them were prospective comparative studies (9, 11, 26–29). In total, 485 affected eyes were analyzed in our study, with 251 high-astigmatism eyes undergoing SMILE surgery, 159 receiving FS-LASIK surgery, 44 taking t-PRK surgery, and 31 low-astigmatism eyes undergoing SMILE surgery (Figure 1). All these studies were published between 2016 and 2022. The demographic characteristics of different groups in each included study were similar. The main characteristics and methodological quality assessment, the details in follow-up, and the outcome variables of the included studies are summarized in Table 1.

**TABLE 1:** Main characteristics of the included studies.

References	Country	Study design	Group design	Group size case/Control (eyes)	Average age case/Control (years)	CCT (mean ± SD)	Cylinder (mean ± SD)	Follow-up (months)	Outcome variables
Zhou et al. (27)	China	P	SMILE/FS-LASIK	53/41	29.79 ± 7.15/27.59 ± 7.47	544.60 ± 24.91/542.66 ± 29.33	−2.51 ± 0.56/−2.65 ± 0.77	12	UDVA/vector
Zhao et al. (26)	China	P	SMILE/FS-LASIK	47/40	20.72 ± 3.81/22.81 ± 5.21	NA	−2.835 ± 0.581 (−4.25 to −1.50)/−2.988 ± 0.679 (−4.50 to −1.50)	3	UDVA/aberration/vector
Zhong et al. (29)	China	P	HA/LA	43/31	24.6 ± 3.9/25.3 ± 4.2	NA	−2.47 ± 0.54/−0.55 ± 0.28	49.5 ± 2.9	UDVA/aberration/vector
Jun et al. (28)	Korea	P	SMILE/t-PRK	45/44	24.80 ± 4.56/25.80 ± 3.47	560.49 ± 29.20 (506 to 641)/555.70 ± 29.01 (509 to 656)	−2.90 ± 0.42 (−4.37 to −2.50)/−2.84 ± 0.35 (−3.75 to −2.50)	6	UDVA/aberration/vector
Zhang et al. (11)	China	P	SMILE/FS-LASIK	23/13	22 ± 5/24 ± 5	NA	−2.48 ± 0.82/−2.56 ± 1.04	NA	Vector
Chan et al. (9)	China	P	SMILE/FS-LASIK	40/65	27.0 ± 5.8/31.4 ± 5.8	541.6 ± 24.8/535.4 ± 25.5	−3.42 ± 0.55/−3.47 ± 0.49	3	UDVA/vector

P, prospective comparative study; SMILE, small-incision lenticule extraction; FS-LASIK, femtosecond laser-assisted laser *in situ* keratomileusis; HA, high astigmatism; LA, low astigmatism; T-PRK, transepithelial photorefractive keratectomy; NA, not available; UDVA, uncorrected distance visual acuity; aberration, corneal aberrations; vector, vector analysis.

### 3.2. Risk of bias assessment

**FIGURE 2:**
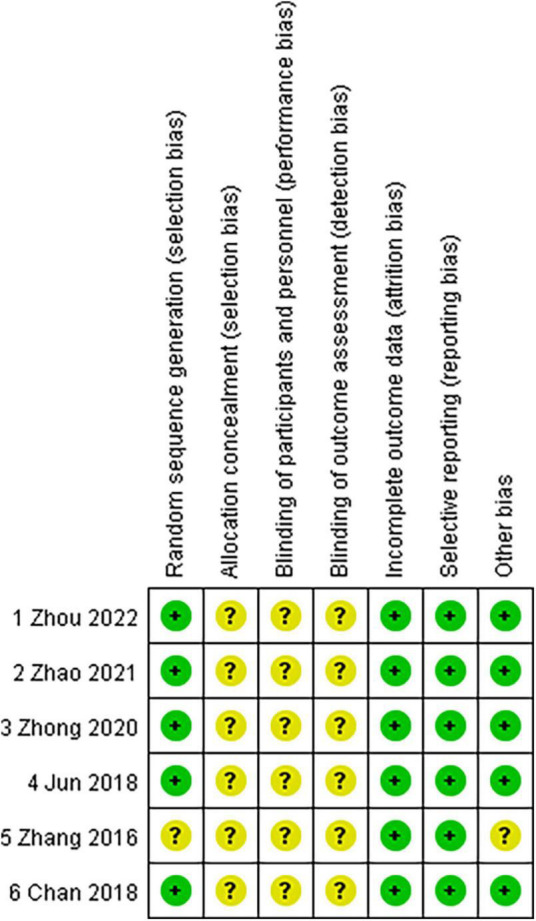
Risk of bias assessment of randomized controlled trials included.

The risk of bias of the six RCTs included is shown in Figure 2 (available in the online version of this article). The random number table method was only applied in one study (26) for random grouping. In clinical practice, it is challenging to completely randomize patients into two groups because those patients with the thin cornea and high myopic astigmatism may not be good SMILE candidates. Except for one study (11) where the decision of choosing surgical strategy was completely left up to the patient, randomization was thought to have been relatively well accomplished. Allocation concealment was not mentioned and was graded as an unclear risk of bias in all studies (9, 11, 26–29). In terms of performance bias and detection bias, all trials were poorly rated as unclear risk of bias (9, 11, 26–29). Regarding attrition bias and reporting bias, all studies were graded as low risk of bias (incomplete outcome data) (9, 11, 26–29). As to other biases, only one study (11) was considered as the unclear risk of bias.

### 3.3. Clinical outcomes

#### 3.3.1. UDVA and residual refractive diopters

**FIGURE 3:**
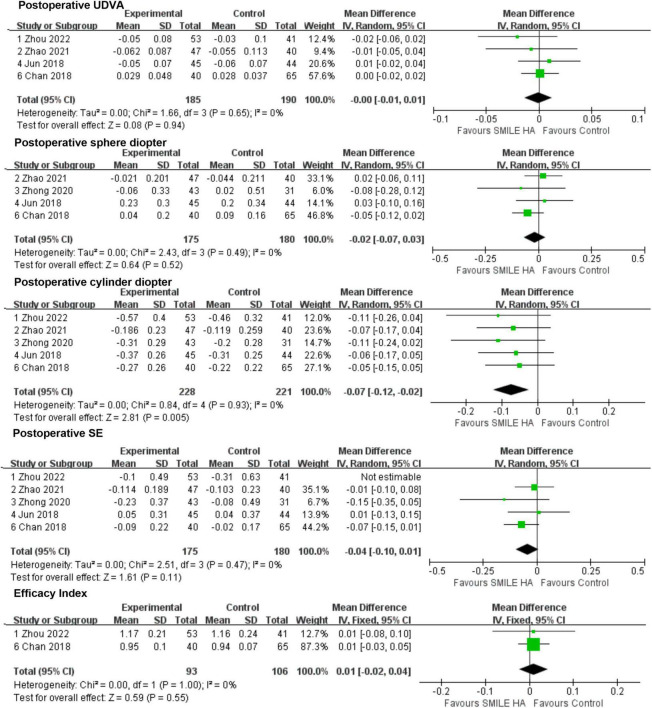
Comparison of visual outcomes and efficacy index between two groups. UDVA, uncorrected distance visual acuity; SE, sphere equivalent.

In all, four publications (9, 26–28) reported the UDVA at the end of follow-up post-operatively. Analysis of the UDVA revealed no statistically significant difference between the SMILE group and the non-SMILE group (WMD = −0.00, 95% CI [−0.01 to 0.01], *P* = 0.94) (Figure 3).

The aforementioned four studies (9, 26, 28, 29) were considered in the results of the sphere diopter. No statistically significant difference between the SMILE and the non-SMILE groups was found (WMD = −0.02, 95% CI [−0.07 to 0.03], *P* = 0.52) (Figure 3). However, the predictability of the correction of spherical refractive error was excellent in both groups.

A total of five included studies (9, 26–29) mentioned the residual astigmatism diopter at the end of follow-up post-operatively. A significant difference between the SMILE and the non-SMILE groups was found (WMD = −0.07, 95% CI [−0.12 to −0.02], *P* = 0.005) (Figure 3). SMILE induced more residual astigmatism than the non-SMILE group.

The five studies (9, 26–29) exhibited residual SE at the end of follow-up post-operatively. However, Zhou’s study (27) demonstrated high heterogeneity, and after removing this study, the *I*^2^ was 0. No statistically significant difference between the SMILE and the non-SMILE group was found (WMD = −0.04, 95% CI [−0.10 to 0.01], *P* = 0.11) (Figure 3).

Among 211 eyes that underwent SMILE surgery, none of them were reported to suffer from any severe ocular or systemic adverse events. No statistically significant difference in efficacy index between the SMILE and non-SMILE groups was found (WMD = 0.01, 95% CI [−0.02 to 0.04], *P* = 0.55) (Figure 3).

#### 3.3.2. Aberrations

**FIGURE 4:**
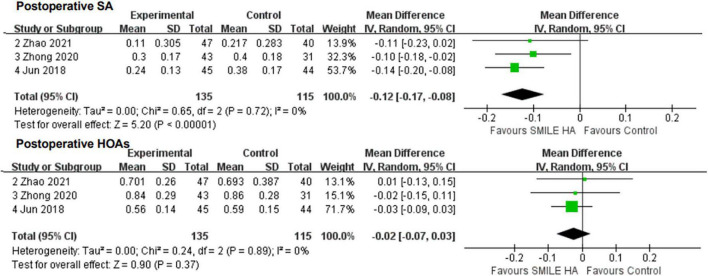
Comparison of visual outcomes and efficacy index between two groups. SA, sphere aberrations; HOAs, higher-order aberrations.

Overall, three studies (26, 28, 29) covered the changes in SA. SMILE led to lesser surgically induced SA than the non-SMILE group, with a statistically significant difference (WMD = −0.12, 95% CI [−0.17 to −0.08], *P* < 0.00001) (Figure 4).

Of the three, two studies (28, 29) reported data for the changes in a coma. No statistically significant difference between the SMILE and the non-SMILE groups was found (WMD = 0.06, 95% CI [−0.04 to 0.16], *P* = 0.27) (Figure 4).

All three studies (26, 28, 29) compared the changes in HOAs. No statistically significant difference between the SMILE and the non-SMILE groups was found (WMD = −0.02, 95% CI [−0.07 to 0.03], *P* = 0.37) (Figure 4).

#### 3.3.3. Vector analysis

Overall, five studies provided the changes in TIA (9, 11, 26–28), SIA (9, 11, 26–28), and ME (9, 11, 27–29), six studies (9, 11, 26–29) exhibited the changes in DV and CI, and four studies (9, 11, 27, 28) were considered for the changes in IOS.

**FIGURE 5:**
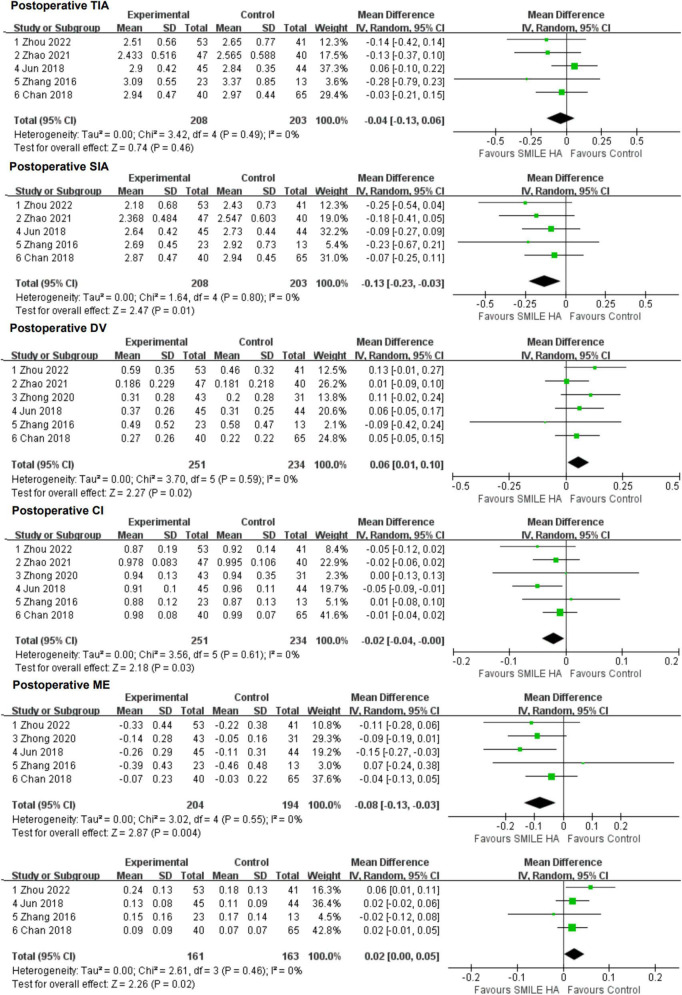
Comparison of visual outcomes and efficacy index between two groups. TIA, vector parameters including target-induced astigmatism vector; SIA, surgically induced astigmatism vector; DV, difference vector; CI, correction index (SIA/TIA); ME, magnitude of error (the arithmetic difference between the magnitudes of the SIA and TIA); IOS, index of success (DV/TIA).

Small-incision lenticule extraction led to lesser SIA and CI than the non-SMILE group, with a statistically significant difference (WMD = −0.13, 95% CI [−0.23 to −0.03], *P* = 0.01; WMD = −0.02, 95% CI [−0.04 to −0.00], *P* = 0.03; respectively) (Figure 5).

Small-incision lenticule extraction led to more DV, ME, and IOS than the non-SMILE group, with a statistically significant difference (WMD = 0.06, 95% CI [0.01 to 0.10], *P* = 0.02; WMD = −0.08, 95% CI [−0.13 to −0.03], *P* = 0.004; WMD = 0.02, 95% CI [0.00 to 0.05], *P* = 0.02; respectively) (Figure 5).

No statistically significant difference in TIA between the SMILE and the non-SMILE groups was found (WMD = −0.04, 95% CI [−0.13 to 0.06], *P* = 0.46) (Figure 5).

## 4. Discussion

With several benefits, SMILE is gaining increasing popularity in treating myopia. In practice, some individuals may be eligible for SMILE, FS-LASIK, and T-PRK, and choices must be made regarding which procedure to perform. The effectiveness of various treatments for high astigmatism ≥2.00 D in clinical myopia correction is debatable. Previous studies had shown that residual astigmatism after SMILE is worsened by the absence of intraoperative pupillary spin correction. After a thorough search was conducted in order to conduct a meta-analysis analyzing the efficacy of SMILE for the treatment of high astigmatism, six pertinent papers comparing the effectiveness of SMILE with other treatments for the correction of high astigmatism were discovered. The evaluation indices of post-operative efficacy were UDVA, residual diopters, aberrations, and astigmatism vector analysis.

Our meta-analysis showed that the efficacy of SMILE and non-SMILE procedures is equivalent. The two groups’ post-operative UDVA, sphere, SE, and HOAs were likewise equivalent. In SMILE, the residual cylinder diopter was larger and the post-operative SA was smaller. Although the DV and IOS were much greater in SMILE, the SIA, CI, and ME were significantly lower, indicating that SMILE has a more cylindrical under-correction. The main source of the heterogeneity in SE was Zhou’s study (27); in this study, the pre-operative SE in FS-LASIK was statistically significantly greater than SMILE, and the post-operative residual SE in FS-LASIK was still higher than SMILE, even though the difference was not statistically significant. Coma and the safety index were only described by two studies, making it impossible to compare the two indices because of significant heterogeneity.

Small-incision lenticule extraction induced more residual astigmatism than the non-SMILE group. The results of our investigation and other studies indicate that the predictability of the astigmatic correction using SMILE is constrained. The optimal post-operative UDVA after refractive surgeries requires precise astigmatic correction (30). In our comparison investigation, the post-operative manifest cylinder magnitudes in the SMILE group were significantly greater than those of the non-SMILE group. This result is in line with earlier studies (12, 13, 15, 31) on the management of low to moderate astigmatism. However, when correcting high astigmatism, both SMILE and FS-LASIK exhibited under-corrections in Zhang’s study (11), although the difference was not statistically significant. Despite the association being poor, there is a tendency for greater under-correction in eyes with a higher astigmatism magnitude (12), which is comparable to the trend following FS-LASIK published in earlier studies (32, 33) that over-correction in low astigmatism and under-correction in high astigmatism. This outcome is in accordance with earlier studies (16, 34, 35) that discovered SMILE was less effective in correcting low or high astigmatism than it was at correcting moderate astigmatism. However, Ganesh and Gupta (36) did not discover any appreciable variations in the post-operative cylinder between SMILE and FS-LASIK.

It was hypothesized that pre-operative attempted astigmatism correction, axis rotation during surgery, or wound healing after surgery could all have an impact on under-correction (31). There is an under-correction of up to 21% of the attempted cylinder correction when utilizing the MEL-80 excimer laser for FS-LASIK to treat high astigmatism (mean 3.9 D) in myopic eyes (32). One of the main causes of astigmatic under-correction with SMILE is the lack of intraoperative torsional control and the lack of control of cyclotorsion. This could be an indication of a varied healing response following various operations (37). The cutting effect within the cornea is achieved by SMILE using a femtosecond laser in the near-infrared band by photodisruption. When compared to the excimer laser, the femtosecond laser delivered far less energy to the cornea (38). Under- or over-ablation of stromal tissue during excimer laser surgery is most likely caused by variations in the corneal stroma’s moisture (36). According to mathematical calculations, SMILE has better tensile strength than PRK and LASIK (39).

The cylinder was higher in the SMILE group in Chan’s study (15), and as a result, the UDVA was poor in the SMILE group. This phenomenon did not occur in our investigation. In contrast to two earlier studies (12, 13, 40), which revealed that the SE in the SMILE group was higher than the non-SMILE group, the SE between the two groups in our study did not differ. The fact that all of the patients in our study have astigmatism more than 2.00 D, as opposed to other studies that may have included patients with astigmatism less than 2.00 D, may account for the difference between our study and previous ones. Less surgically induced SA was caused by SMILE than by the non-SMILE group. Further research by Lin et al. (40) showed that SMILE and FS-LASIK have different induction rates for HOAs and SA. These investigations showed no change in a coma, whereas SMILE generally had considerably less SA induction, indicating that it preserves corneal asphericity better than FS-LASIK (40–42). The increase in spherical aberration of the FS-LASIK may be due to the cosine effect of the excimer laser (26).

The magnitude of inaccuracy was much larger in the SMILE group in our series, which is consistent with earlier research (12) and supports the existence of some degree of under-correction. In addition, SIA was lower and the estimated CI was less than 1, indicating that astigmatism under-corrected following SMILE. When the pre-operative astigmatism was greater than 1.0 D, both SMILE and Laser-Assisted Subepithelial Keratomileusis (LASEK) in Qian’s study (16) showed an under-correction, and the under-correction grew larger with the degree of the TIA. The outcomes were equivalent to those of existing studies (31, 35). The DV was significantly higher in the SMILE group compared to the non-SMILE group, confirming that there was a greater astigmatism correction deviation from the target with SMILE. This finding is consistent with those made by Chan et al. (15) and Khalifa (12), who found that SMILE provides less effective astigmatism correction than FS-LASIK. When compared to LASIK, SMILE had less “success” in treating astigmatism since post-operative astigmatism or DV as well as the IOS was higher. There is a much greater deviation of the surgically induced axis from the target in eyes receiving SMILE because the angle of error after SMILE was significantly higher in the SMILE group than in the FS-LASIK group in earlier studies (12, 15). Studies (36, 40) contrasting FS-LASIK and SMILE revealed that SMILE might offer better visual results. In addition, the safety and predictability were noticeably improved following SMILE, as seen by an increase in CDVA and post-operative refractive error. Lin et al. (40) also noted a trend toward a higher efficacy index in SMILE. The efficacy index did not significantly differ between the two groups, according to Ganesh and Gupta’s study (36). In our investigation, there was no difference in the efficacy index between the groups.

The study’s limitation is the lack of studies comparing the efficacy of different surgeries for high astigmatism; consequently, the studies that were included in the analysis were few and pertinent. Subsequent clinical observational studies can compare the effectiveness analyses of SMILE and FS-LASIK for high astigmatism. The degree of astigmatism included in previous studies did not have a set standard, varying from 0.25 to 4.00 D; the following studies researching the effectiveness of treating astigmatism could adhere to a fixed standard. Looking forward to the future of VisuMax, we believe that a device with cyclotorsion control could help physicians correct high astigmatism more accurately.

This study suggested that SMILE, FS-LASIK, and T-PRK had comparable efficacy and safety in correcting high myopic astigmatism (≥2.00 D). SMILE induced more residual astigmatism than the non-SMILE group. SMILE may have smaller post-operative SA, smaller SIA, and larger DV. Astigmatism under-correction occurred more frequently in SMILE.

## Data availability statement

The raw data supporting the conclusions of this article will be made available by the authors, without undue reservation.

## Ethics statement

The studies involving human participants were reviewed and approved by the Institutional Review Board/Ethics Committee of PUMCH. The patients/participants provided their written informed consent to participate in this study.

## Author contributions

GC conceived this study and wrote the draft of the manuscript. GC and YD conducted the data collection and analysis. GC and SY assessed the quality of the included studies. DC and YL provided guidance as a third researcher when there was a disagreement. YD, SY, and DC assisted in the draft. YL conducted and coordinated the whole process. All authors have read the final manuscript and reached an agreement.

## Footnotes

1
http://www.aaojournal.org/

## References

[1] Sekundo W Kunert K Russmann C Gille A Bissmann W Stobrawa G First efficacy and safety study of femtosecond lenticule extraction for the correction of myopia: six-month results. *J Cataract Refract Surg.* (2008) 34:1513–20. 10.1016/j.jcrs.2008.05.033 18721712

[2] Reinstein D Archer T Gobbe M. Small incision lenticule extraction (SMILE) history, fundamentals of a new refractive surgery technique and clinical outcomes. *Eye Vis.* (2014) 1:3. 10.1186/s40662-014-0003-1 26605350 PMC4604118

[3] Shah R. History and results; indications and contraindications of smile compared with LASIK. *Asia Pac J Ophthalmol.* (2019) 8:371–6. 10.1097/01.APO.0000580132.98159.fa PMC6784775 31567264

[4] Yang L Mehta J Liu Y. Corneal neuromediator profiles following laser refractive surgery. *Neural Regen Res.* (2021) 16:2177–83. 10.4103/1673-5374.308666 33818490 PMC8354117

[5] Guo H Hosseini-Moghaddam S Hodge W. Corneal biomechanical properties after smile versus FLEX, LASIK, LASEK, or PRK: a systematic review and meta-analysis. *BMC Ophthalmol.* (2019) 19:167. 10.1186/s12886-019-1165-3 31370817 PMC6676534

[6] Arbelaez M Aslanides I Barraquer C Carones F Feuermannova A Neuhann T LASIK for myopia and astigmatism using the SCHWIND AMARIS excimer laser: an international multicenter trial. *J Refract Surg.* (2010) 26:88–98. 10.3928/1081597X-20100121-04 20163073

[7] Vestergaard A Ivarsen A Asp S Hjortdal J. Small-incision lenticule extraction for moderate to high myopia: Predictability, safety, and patient satisfaction. *J Cataract Refract Surg.* (2012) 38:2003–10. 10.1016/j.jcrs.2012.07.021 22981612

[8] Alpins N. Astigmatism analysis by the alpins method. *J Cataract Refract Surg.* (2001) 27:31–49. 10.1016/S0886-3350(00)00798-7 11165856

[9] Chan T Wang Y Ng A Zhang J Yu M Jhanji V Vector analysis of high (=3 diopters) astigmatism correction using small-incision lenticule extraction and laser in situ keratomileusis. *J Cataract Refract Surg.* (2018) 44:802–10. 10.1016/j.jcrs.2018.04.038 29909252

[10] Pedersen I Ivarsen A Hjortdal J. Changes in astigmatism, densitometry, and aberrations after smile for low to high myopic astigmatism: A 12-month prospective study. *J Refract Surg.* (2017) 33:11–7. 10.3928/1081597X-20161006-04 28068441

[11] Zhang J Wang Y Chen X. Comparison of moderate- to high-astigmatism corrections using wavefront-guided laser in situ keratomileusis and small-incision lenticule extraction. *Cornea.* (2016) 35:523–30. 10.1097/ICO.0000000000000782 26890662

[12] Khalifa M Ghoneim A Shaheen M Piñero D. Vector analysis of astigmatic changes after small-incision lenticule extraction and wavefront-guided laser in situ keratomileusis. *J Cataract Refract Surg.* (2017) 43:819–24. 10.1016/j.jcrs.2017.03.033 28732617

[13] Kanellopoulos A. Topography-guided LASIK versus small incision lenticule extraction (smile) for myopia and myopic astigmatism: a randomized, prospective, contralateral eye study. *J Refract Surg.* (2017) 33:306–12. 10.3928/1081597X-20170221-01 28486721

[14] Alió Del Barrio J Vargas V Al-Shymali O Alió J. Small incision lenticule extraction (SMILE) in the correction of myopic astigmatism: outcomes and limitations - an update. *Eye Vis.* (2017) 4:26. 10.1186/s40662-017-0091-9 29167808 PMC5686829

[15] Chan T Ng A Cheng G Wang Z Ye C Woo V Vector analysis of astigmatic correction after small-incision lenticule extraction and femtosecond-assisted LASIK for low to moderate myopic astigmatism. *Br J Ophthalmol.* (2016) 100:553–9. 10.1136/bjophthalmol-2015-307238 26206791

[16] Qian Y Huang J Zhou X Wang Y. Comparison of femtosecond laser small-incision lenticule extraction and laser-assisted subepithelial keratectomy to correct myopic astigmatism. *J Cataract Refract Surg.* (2015) 41:2476–86. 10.1016/j.jcrs.2015.05.043 26703499

[17] Chan T Yu M Ng A Wang Z Cheng G Jhanji V. Early outcomes after small incision lenticule extraction and photorefractive keratectomy for correction of high myopia. *Sci Rep.* (2016) 6:32820. 10.1038/srep32820 27601090 PMC5013393

[18] Moher D Liberati A Tetzlaff J Altman D. Preferred reporting items for systematic reviews and meta-analyses: the PRISMA statement. *J Clin Epidemiol.* (2009) 62:1006–12. 10.1016/j.jclinepi.2009.06.005 19631508

[19] Gregori N Feuer W Rosenfeld P. Novel method for analyzing snellen visual acuity measurements. *Retina.* (2010) 30:1046–50. 10.1097/IAE.0b013e3181d87e04 20559157

[20] Sterne J Savović J Page M Elbers R Blencowe N Boutron I RoB 2: a revised tool for assessing risk of bias in randomised trials. *BMJ.* (2019) 366:l4898. 10.1136/bmj.l4898 31462531

[21] Puhan M Schünemann H Murad M Li T Brignardello-Petersen R Singh J A grade working group approach for rating the quality of treatment effect estimates from network meta-analysis. *BMJ.* (2014) 349:g5630. 10.1136/bmj.g5630 25252733

[22] Higgins J Thompson S Deeks J Altman D. Measuring inconsistency in meta-analyses. *BMJ.* (2003) 327:557–60. 10.1136/bmj.327.7414.557 12958120 PMC192859

[23] Higgins J. Commentary: Heterogeneity in meta-analysis should be expected and appropriately quantified. *Int J Epidemiol.* (2008) 37:1158–60. 10.1093/ije/dyn204 18832388

[24] Borenstein M Hedges L Higgins J Rothstein HR. A basic introduction to fixed-effect and random-effects models for meta-analysis. *Res Synth Methods.* (2010) 1:97–111. 26061376 10.1002/jrsm.12

[25] Riley R Higgins J Deeks J. Interpretation of random effects meta-analyses. *BMJ.* (2011) 342:d549. 10.1136/bmj.d549 21310794

[26] Zhao X Zhang L Ma J Li M Zhang J Zhao X Comparison of wavefront-guided femtosecond LASIK and optimized smile for correction of moderate-to-high astigmatism. *J Refract Surg.* (2021) 37:166–73. 10.3928/1081597X-20201230-01 34038300

[27] Zhou J Gu W Gao Y He G Zhang F. Vector analysis of high astigmatism (≥2.0 diopters) correction after small-incision lenticule extraction with stringent head positioning and femtosecond laser-assisted laser in situ keratomileusis with compensation of cyclotorsion. *BMC Ophthalmol.* (2022) 22:157. 10.1186/s12886-022-02384-0 35382779 PMC8985270

[28] Jun I Kang D Reinstein D Arba-Mosquera S Archer T Seo K Clinical outcomes of smile with a triple centration technique and corneal wavefront-guided transepithelial prk in high astigmatism. *J Refract Surg.* (2018) 34:156–63. 10.3928/1081597X-20180104-03 29522224

[29] Zhong Y Li M Han T Fu D Zhou X. Four-year outcomes of small incision lenticule extraction (SMILE) to correct high myopic astigmatism. *Br J Ophthalmol.* (2021) 105:27–31. 10.1136/bjophthalmol-2019-315619 32201375

[30] Wolffsohn J Bhogal G Shah S. Effect of uncorrected astigmatism on vision. *J Cataract Refract Surg.* (2011) 37:454–60. 10.1016/j.jcrs.2010.09.022 21333869

[31] Zhang J Wang Y Wu W Xu L Li X Dou R. Vector analysis of low to moderate astigmatism with small incision lenticule extraction (SMILE): results of a 1-year follow-up. *BMC Ophthalmol.* (2015) 15:8. 10.1186/1471-2415-15-8 25618419 PMC4328987

[32] Ivarsen A Næser K Hjortdal J. Laser in situ keratomileusis for high astigmatism in myopic and hyperopic eyes. *J Cataract Refract Surg.* (2013) 39:74–80. 23158679 10.1016/j.jcrs.2012.08.054

[33] Frings A Katz T Richard G Druchkiv V Linke S. Efficacy and predictability of laser in situ keratomileusis for low astigmatism of 0.75 diopter or less. *J Cataract Refract Surg.* (2013) 39:366–77. 10.1016/j.jcrs.2012.09.024 23506918

[34] Ivarsen A Hjortdal J. Correction of myopic astigmatism with small incision lenticule extraction. *J Refract Surg.* (2014) 30:240–7. 10.3928/1081597X-20140320-02 24702575

[35] Kunert K Russmann C Blum M Sluyterman V. Vector analysis of myopic astigmatism corrected by femtosecond refractive lenticule extraction. *J Cataract Refract Surg.* (2013) 39:759–69. 23608569 10.1016/j.jcrs.2012.11.033

[36] Ganesh S Gupta R. Comparison of visual and refractive outcomes following femtosecond laser- assisted lasik with smile in patients with myopia or myopic astigmatism. *J Refract Surg.* (2014) 30:590–6. 25250415 10.3928/1081597X-20140814-02

[37] Wei S Wang Y Wu D Zu P Zhang H Su X. Ultrastructural changes and corneal wound healing after SMILE and PRK procedures. *Curr Eye Res.* (2016) 41:1316–25. 10.3109/02713683.2015.1114653 26863271

[38] Riau A Angunawela R Chaurasia S Lee W Tan D Mehta J. Early corneal wound healing and inflammatory responses after refractive lenticule extraction (ReLEx). *Invest Ophthalmol Vis Sci.* (2011) 52:6213–21. 10.1167/iovs.11-7439 21666235

[39] Reinstein D Archer T Randleman J. Mathematical model to compare the relative tensile strength of the cornea after PRK, LASIK, and small incision lenticule extraction. *J Refract Surg.* (2013) 29:454–60. 23820227 10.3928/1081597X-20130617-03

[40] Lin F Xu Y Yang Y. Comparison of the visual results after SMILE and femtosecond laser-assisted LASIK for myopia. *J Refract Surg.* (2014) 30:248–54. 10.3928/1081597X-20140320-03 24702576

[41] Gyldenkerne A Ivarsen A Hjortdal J. Comparison of corneal shape changes and aberrations induced By FS-LASIK and SMILE for myopia. *J Refract Surg.* (2015) 31:223–9. 10.3928/1081597X-20150303-01 25751842

[42] Liu M Chen Y Wang D Zhou Y Zhang X He J Clinical outcomes after Smile and femtosecond laser-assisted LASIK for myopia and myopic astigmatism: a prospective randomized comparative study. *Cornea.* (2016) 35:210–6. 10.1097/ICO.0000000000000707 26684046

