# Supplementary material for: Efficacy of small-incision lenticule extraction surgery in high astigmatism: A meta-analysis
Source: Front Med (Lausanne). 2023 Jan 19;9:1100241. doi: 10.3389/fmed.2022.1100241 (PMC9892059; doi:10.3389/fmed.2022.1100241)
Supplement: Supplementary file 1 [file Table_1.docx]

Efficacy of small incision lenticule extraction surgery in high astigmatism: a meta-analysis

Supplementary Material

Following the PICOS principle, the key search terms included (P, participants) patients with moderate to high myopia; (I, interventions) patients treated by SMILE or other refractive surgeries; (C/O, comparison/outcome) the comparison of the clinical outcomes; and (S, study design) designed as a clinical cohort study.

| P: | “astigmatism” [Mesh] OR high astigmatism OR Moderate-to-High Astigmatism |
| --- | --- |
| I: | “Keratectomy, Subepithelial, Laser-Assisted” [Mesh] OR Laser-Assisted Subepithelial Keratectomy OR Laser Epithelial Keratomileusis OR Laser Subepithelial Keratomileusis OR Laser-Assisted Subepithelial Keratomileusis OR LASEK  “Keratomileusis, Laser In Situ” [Mesh] OR Laser In Situ Keratomileusis OR Laser-assisted in-situ keratomileusis OR epipolis laser in situ keratomileusis OR LASIK OR Epipolis laser in situ keratomileusis OR Epi-LASIK  “Photorefractive Keratectomy” [Mesh] OR Photorefractive Keratectomy OR PRK OR TransPRK OR transepithelial PRK OR transepithelial photorefractive keratectomy  femtosecond lenticule extraction OR Femtosecond laser-assisted laser in-situ keratomileusis OR Flex OR small incision lenticule extraction OR SMILE  Sub Bowman Keratomileusis OR SBK OR refractive surgery OR laser surgery |
| C/O: | “Vision, Ocular” [Mesh] OR Vision OR Ocular Vision OR Light Signal Transduction, Visual OR Visual Light Signal Transduction OR Visual Transduction OR Transduction, Visual OR Visual Phototransduction OR Phototransduction, Visual  “Night Vision” [Mesh] OR Vision, Night OR Rod Vision OR Vision, Rod OR Scotopic Vision OR Vision, Scotopic  “Corneal Wavefront Aberration” [Mesh] OR Aberration, Corneal Wavefront OR Aberrations, Corneal Wavefront OR Corneal Wavefront Aberrations OR Wavefront Aberration, Corneal OR Wavefront Aberrations, Corneal |
| S: | (randomized controlled trial [pt] OR controlled clinical trial [pt] OR randomized [tiab] OR placebo [tiab] OR clinical trials as topic [mesh: noexp] OR randomly [tiab] OR trial [ti]) |

Search closed: June 30^th^, 2022, Language limits: none

Search strategy was designed based on the recommendations of Paisley et al.^1^

The final searching strategy were (“Keratectomy, Subepithelial, Laser-Assisted”[Mesh] OR Laser-Assisted Subepithelial Keratectomy OR Laser Epithelial Keratomileusis OR Laser Subepithelial Keratomileusis OR Laser-Assisted Subepithelial Keratomileusis OR LASEK OR “Keratomileusis, Laser In Situ”[Mesh] OR Laser In Situ Keratomileusis OR Laser-assisted in-situ keratomileusis OR epipolis laser in situ keratomileusis OR LASIK OR Epipolis laser in situ keratomileusis OR Epi-LASIK OR “Photorefractive Keratectomy”[Mesh] OR Photorefractive Keratectomy OR PRK OR TransPRK OR transepithelial PRK OR transepithelial photorefractive keratectomy OR femtosecond lenticule extraction OR Femtosecond laser-assisted laser in-situ keratomileusis OR Flex OR small incision lenticule extraction OR SMILE OR Sub Bowman Keratomileusis OR SBK OR refractive surgery OR laser surgery) AND (randomized controlled trial [pt] OR controlled clinical trial [pt] OR randomized [tiab] OR placebo [tiab] OR clinical trials as topic [mesh: noexp] OR randomly [tiab] OR trial [ti]) AND (“astigmatism”[Mesh] OR high astigmatism OR Moderate-to-High Astigmatism) AND (“Vision, Ocular”[Mesh] OR Vision OR Ocular Vision OR Light Signal Transduction, Visual OR Visual Light Signal Transduction OR Visual Transduction OR Transduction, Visual OR Visual Phototransduction OR Phototransduction, Visual OR “Night Vision”[Mesh] OR Vision, Night OR Rod Vision OR Vision, Rod OR Scotopic Vision OR Vision, Scotopic OR “Corneal Wavefront Aberration”[Mesh] OR Aberration, Corneal Wavefront OR Aberrations, Corneal Wavefront OR Corneal Wavefront Aberrations OR Wavefront Aberration, Corneal OR Wavefront Aberrations, Corneal).

1. Paisley S BA, Mensinkai S. Health-related Quality of Life Studies <http://wwwnlmnihgov/archive/20060905/nichsr/ehta/chapter12html> Accessed: 11/12/2014 2000.
